# Shape tailoring of AgBr microstructures: effect of the cations of different bromide sources and applied surfactants†

**DOI:** 10.1039/d0ra09144h

**Published:** 2021-03-09

**Authors:** Zsejke-Réka Tóth, Zsolt Pap, János Kiss, Lucian Baia, Tamás Gyulavári, Zsolt Czekes, Milica Todea, Klára Magyari, Gábor Kovács, Klara Hernadi

**Affiliations:** Department of Applied and Environmental Chemistry, University of Szeged Rerrich Béla tér 1 HU-6720 Szeged Hungary k.gabor84@chem.u-szeged.hu; Nanostructured Materials and Bio-Nano-Interfaces Center, Institute for Interdisciplinary Research on Bio-Nano-Sciences, Babeş-Bolyai University Treboniu Laurian 42 RO-400271 Cluj-Napoca Romania gkovacs@chem.ubbcluj.ro; Institute of Environmental Science and Technology, University of Szeged Tisza Lajos krt. 103 HU-6720 Szeged Hungary; Institute of Research-Development-Innovation in Applied Natural Sciences, Babes-Bolyai University Fântânele 30 RO-400294 Cluj-Napoca Romania; Faculty of Physics, Babeş-Bolyai University M. Kogălniceanu 1 RO-400084 Cluj-Napoca Romania; Hungarian Department of Biology and Ecology, Babeş-Bolyai University Clinicilor 5–7 RO-400006 Cluj-Napoca Romania; Iuliu Hatieganu University of Medicine and Pharmacy, Faculty of Medicine Victor Babeş 8 RO-400012 Cluj-Napoca Romania; Institute of Physical Metallurgy, Metal Forming and Nanotechnology, University of Miskolc 3515 Miskolc-Egyetemváros Hungary

## Abstract

Investigations regarding AgBr-based photocatalysts came to the center of attention due to their high photosensitivity. The present research focuses on the systematic investigation regarding the effect of different alkali metal cation radii and surfactants/capping agents applied during the synthesis of silver-halides. Their morpho-structural and optical properties were determined *via* X-ray diffractometry, diffuse reflectance spectroscopy, scanning electron microscopy, infrared spectroscopy, and contact angle measurements. The semiconductors' photocatalytic activities were investigated using methyl orange as the model contaminant under visible light irradiation. The correlation between the photocatalytic activity and the obtained optical and morpho-structural properties was analyzed using generalized linear models. Moreover, since the (photo)stability of Ag-based photoactive materials is a crucial issue, the stability of catalysts was also investigated after the degradation process. It was concluded that (i) the photoactivity of the samples could be fine-tuned using different precursors and surfactants, (ii) the as-obtained AgBr microcrystals were transformed into other Ag-containing composites during/after the degradation, and (iii) elemental bromide did not form during the degradation process. Thus, the proposed mechanisms in the literature (for the degradation of MO using AgBr) must be reconsidered.

## Abstract 2

Systematic investigation of the effect of different alkali metal cation radii and shape-tailoring agents applied during the synthesis of AgBr-based photocatalysts.

## Graphical abstract

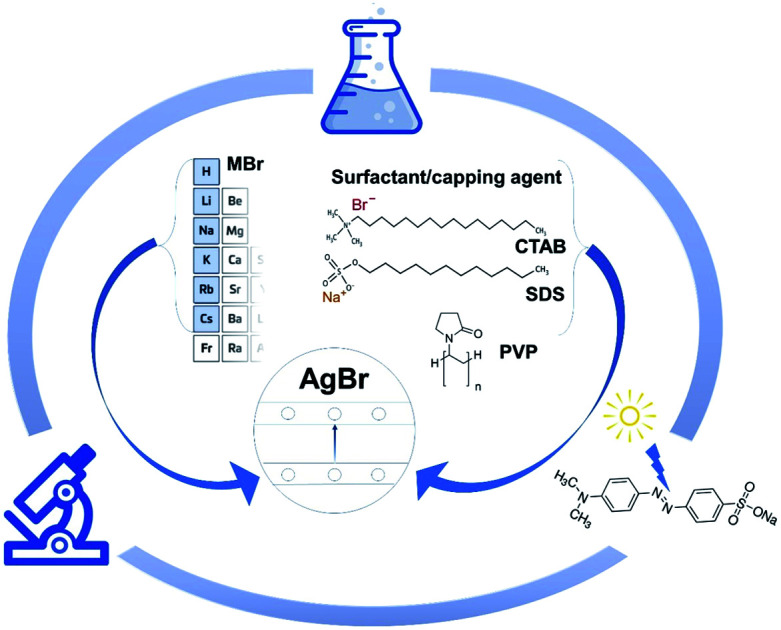

## Introduction

The renewed interest toward silver-based semiconductors is not surprising. The applicability of Ag nanoparticles is well-known even from ancient times due to their antibacterial character; however, their practical applications were only popular in the 1900s.^1^ Moreover, due to their low stability (formation of silver nanoparticles on their surface), the applicability of silver-containing semiconductors is still low. Nevertheless, they are excitable under visible light irradiation (having a relatively narrow band gap energy, *e.g.*, Ag_2_O: 1.2 eV;^2^ Ag_2_S: 0.9–1.0 eV;^3^ and Ag_3_PO_4_: 2.43 eV (ref. 4)) and can be synthesized easily. There is still a dispute regarding whether their instability is an advantage or a disadvantage; by noble metal deposition, although the structure and properties change, they are usually beneficial.^5^

One of the most interesting silver-based materials is Ag_2_O, a p-type semiconductor with relatively low stability. Due to its low stability, it disproportionates under visible light irradiation and gives Ag and AgO.^2^ Another interesting material is Ag_2_S, an n-type semiconductor with a large visible light absorption coefficient,^6^ showing luminescent properties.^7^ Because of the low stability of the semiconductors mentioned above, other Ag-based photocatalytic materials have been investigated, such as Ag_3_PO_4_,^8^ Ag_2_SO_4_,^9^ Ag_2_CO_3_,^10^ and delafossite-type Ag-based semiconductors (*e.g.*, AgGaO_2_ (ref. 11) or AgAlO_2_ (ref. 12)). Moreover, the affinity of Ag-based materials for photocorrosion could be decreased using the composites of two Ag-based semiconductors such as Ag_2_O/Ag_2_CO_3_,^13^ Ag_2_S/Ag_2_WO_4_,^14^ Ag_2_S@Ag_2_CO_3_,^15^ AgCl/Ag_2_CO_3_,^16,17^ AgBr/AgIO_3_,^18^ and Ag_3_PO_4_@AgBr.^19^

Silver halides also appeared in different applications, including photographic techniques.^20^ Moreover, silver halides are more prevalent in photocatalytic processes (*e.g.*, AgCl,^21^ AgBr,^22^ and AgI^23^). Silver halides usually have narrow band gap energy (about 3.2 eV for chlorides,^24^ 2.6 eV for bromides,^25^ and 2.8 eV for iodides^23^), can be synthesized rather simply (*e.g.*, by ion exchange,^26^ precipitation,^27^ or hydrothermal crystallization processes^28^), and possess relatively increased photosensitivity. Among these types of halides, silver bromide is one of the most widely used as a photocatalyst.^26^ Also, in the case of AgBr, silver nanoparticles/nanoclusters can be formed during photocatalytic processes.^29^ Interestingly, the as-formed Ag nanoclusters can be selectively adsorbed on the (110) crystallographic plane of AgBr, according to a theoretical calculation.^29^ Therefore, researchers working in this field have been focusing on manipulating the (111)/(110) ratio to control the amount of the as-formed and deposited Ag.

Moreover, the amount of the deposited/formed Ag nanoparticles as essential as the obtained shape of the photocatalyst since AgBr octahedra with exposed (111) facets showed higher activity than cubes and spheres.^30^

One approach to control the shape of the catalyst could be the usage of surfactants/shape-tailoring agents during the synthesis since, depending on their structure, the morphology and size of the semiconductor crystal can be controlled.^31^ The most-studied shape-tailoring/capping agent is polyvinylpyrrolidone (PVP), which is a polymer (monomer, *N*-vinylpyrrolidone) and a non-ionic surfactant at the same time. Sodium dodecyl sulfate (SDS) and cetyltrimethylammonium bromide (CTAB) are among the most widely-applied surfactants. SDS is an anionic,^32^ while CTAB is a cationic surfactant, both with broad applicability spectra, which have already been used simultaneously.^33^

Differently shaped AgBr microcrystals have already been synthesized using different surfactants/shape-tailoring agents, such as PVP^34,35^ and CTAB (which can act as a shape-tailoring agent and can be used as bromide source as well^28,36^). In many cases, PVP is used as a capping agent to increase the formation of the (111) crystallographic plane,^34^ thereby increasing the number of edges and corners with specific morphologies, such as polyhedral,^28^ nanorods,^37^ and hollow cubic.^38^ Moreover, it can be used to influence the primary crystallite size.^39^

Until now, to the best of the authors' knowledge, there is no available data/research concerning the application of SDS as a shape-tailoring agent in the case of AgBr. However, there have been reports about the synthesis of Ag_2_S where SDS has been applied successfully.^40^ In several other cases,^41,42^ SDS has been used as an anionic surfactant in the synthesis of semiconductors with high monodispersity. Furthermore, even if CTAB is mainly considered as a surfactant, AgBr microcrystals can be obtained using CTAB as a bromide source,^28^ using precipitation,^36^ ion exchange,^43^ and hydrothermal^44^ methods.

Besides CTAB, different alkali metals, such as sodium^45^ and potassium^38^ ions are used as alkali metal-based Br sources to synthesize AgBr microcrystals. Moreover, the alkali metal cations could be incorporated in the structure of AgBr, creating interstitial defects in the surface.^46^

Accordingly, the current work's main aim was to systematically investigate the effect of different surfactants/capping agents and alkali metal-based Br sources on the morpho-structural, optical, and stability parameters of AgBr-based materials. To the best of our knowledge, no such investigation has been conducted so far in the literature. CTAB, SDS, and PVP were used as surfactants/capping agents, while H^+^, Li^+^, Na^+^, K^+^, Rb^+^, and Cs^+^ were used as the Br sources' cations.

## Experimental

### Materials

The chemicals were used as purchased without further purification. Ethylene glycol (EG, analytical reagent) and ethanol (EtOH, analytical reagent) were purchased from Molar Chemicals (Hungary). The applied bromide sources were as follows: hydrobromic acid (HBr, 47–49%, Alfa Aesar (Germany)); anhydrous lithium bromide (LiBr, >99%, Alfa Aesar (Germany)); sodium bromide (NaBr, analytical reagent, Reanal (Hungary)); potassium bromide (KBr, analytical reagent, Reanal (Hungary)); rubidium bromide (RbBr, 99.8%, metal basis, Alfa Aesar (Germany)); cesium bromide (CsBr, 99%, metal basis, Alfa Aesar (Germany)). Silver nitrate (AgNO_3_, analytical reagent) was purchased from the Penta industry (Romania). The applied shape tailoring agents were as follows: cetyltrimethylammonium bromide (CTAB, >98%, Sigma-Aldrich (Steinheim, Germany)); sodium dodecyl sulfate (SDS, ReagentPlus, Biolab (Hungary)); polyvinylpyrrolidone (PVP, average molecular weight 40 000, Sigma-Aldrich (Steinheim, Germany)). It should be mentioned that the first two (CTAB and SDS) are considered by the literature as surfactants, while PVP is used as a capping agent (this is the reason why the term surfactants/capping agent is used throughout the manuscript). Methyl orange (MO, analytical reagent) was used as a model contaminant, which was acquired from Alfa Aesar (Germany).

In this work, the investigated alkali metal elements (Li^+^, Na^+^, K^+^, Rb^+^, and Cs^+^) together with H^+^/the corresponding acid (HBr) will be abbreviated as “S1 chemical elements”.

### Solvothermal synthesis of AgBr photocatalysts

AgBr photocatalysts were synthesized *via* a solvothermal synthetic route.^28^ In the first step, two solutions were prepared—“solution A” contained 100 mL of EG, different amounts of halide sources (varied based on the different molecular weights), and 0.4 g surfactant. “Solution B” contained 20 mL EG and 0.570 g AgNO_3_. Alkali metal salts with different cationic radii (Li^+^, Na^+^, K^+^, Rb^+^, and Cs^+^) and the corresponding acid (HBr) were used to optimize the photocatalysts. The molar ratio of Ag : Br was 1 : 0.42 in each case. Different capping agents/surfactants were used (polyvinylpyrrolidone – PVP, sodium dodecyl sulfate – SDS, and cetyltrimethylammonium bromide – CTAB) to facilitate the formation of monodisperse particles. Also, a reference sample was synthesized without using additives, which was denoted as – NØ.

Solution A was kept at 60 °C for 1 h under vigorous stirring. After this process, solution B was added into solution A; then, an immediate color change from transparent to green/greenish-yellow was observed. The as-obtained synthetic mixture was kept at 60 °C for 1 h. Then, it was transferred into a Teflon®-lined autoclave (160 mL) and kept at 160 °C for 2 h. After the crystallization process, the synthetic mixture was cooled down to room temperature. The solid product was then washed and centrifuged with 3× ≈50 mL H_2_O and 1× ≈25 mL EtOH for 10 min at 4400 RPM. After the cleaning process, the solid product was dried for 12 h at 40 °C. The obtained photocatalysts were denoted as follows: AgBr_MBr_S, where M is the alkali metal (Li^+^, Na^+^, K^+^, Rb^+^, and Cs^+^) or H^+^, where S is the used surfactant/capping agent (PVP, SDS, CTAB, and NØ).

### Characterization of the methods and instrumentation

A Rigaku Miniflex II X-ray diffractometer (XRD) was used for the structural characterization at *λ*_CuKα_ = 0.15406 nm, 40 kV, and 30 mA as the instrument parameters in the range of 20–50° (2*θ*°) with a scanning speed of 1 (2*θ*°) min^−1^. The Scherrer equation was used for the calculation of the mean primary crystallite size.^47^

A Hitachi S-4700 Type II scanning electron microscope (SEM) was used to determine the samples' particle sizes. For electron beam production and acceleration, a cold field emission gun and 10 kV acceleration voltage were applied. The morphology was observed by collecting the secondary electrons with an Everhart-Thornley detector.

A JASCO-V650 spectrophotometer, equipped with an ILV-724 integration sphere, was used for acquiring information about the optical properties of the photocatalysts. The spectra of the samples were recorded between 250–800 nm and the indirect band gap energies were calculated using the Kubelka–Munk equation.^48,49^

Surface tension measurements were carried out using a stalagmometer (*V* = 3.5 mL), applying Milli-Q water as the reference solution. The solutions' density was determined using a pycnometer (*V* = 10 mL) at 25–26 °C. The surface tension values were determined using the following equation
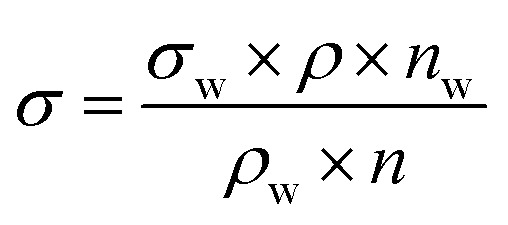
where *σ*, *σ*_w_ are surface tension values (mN m^−1^); *n*, *n*_w_ are the numbers of the counted liquid drops; *ρ*, *ρ*_w_ are the density values of the liquids (g cm^−3^); and *w* in the subscript stands for water.

The samples were investigated by IR spectroscopy using a Jasco 6000 (Jasco, Tokyo, Japan) spectrometer in the range 400–4000 cm^−1^ with a spectral resolution of 4 cm^−1^. The collected samples were centrifuged and dried for 12 h at 40 °C. The dried samples were added to KBr powder to produce the pellets. The possible presence of surfactants was also investigated.

The hydrophilicity of the catalysts was evaluated with a Dataphysics O.C.A. 15EC type optical contact angle meter (using the Dataphysics Contact Angle System OCA15Pro software). Small pellets were prepared using ≈200 mg of the photocatalyst powder, while 10 μL of water was used to measure the contact angle.

The photocatalytic performance was investigated by the degradation of 125 μM methyl orange solution. A double-walled photoreactor (100 mL) was thermostated by 1 M NaNO_2_ solution (to eliminate any ultraviolet (UV) photons) and irradiated by 4 × 24 W (DÜVI 25920/R7S, Hungary, *λ*_max_ = 545 nm) visible light lamps. During the experiments, continuous airflow and stirring were applied. The concentration of the suspension was 1 g L^−1^. The system was kept in the dark for 10 min to reach adsorption–desorption equilibrium, followed by sampling in the first one hour in 10 minute intervals and in the second hour in 20 minute intervals. The obtained samples were centrifugated at 13 400 rpm for 3 min and then filtered using a Whatman Anotop Syringe Filter. An Agilent 8453 spectrophotometer was applied to determine the concentration of methyl orange (*λ*_det_ = 464 nm) using a 0.2 mm optical quartz cuvette.

It is worth mentioning that adsorption occurred in some cases. The adsorption of MO was negligible for AgBr_CsBr_NØ, AgBr_LiBr_PVP, and AgBr_KBr_SDS (Fig. S1†). The highest adsorption value (Fig. S1†) was obtained for AgBr_NaBr_CTAB (20% adsorption of MO). AgBr_CsBr_CTAB showed enhanced adsorption (100%) of MO during ultrasonication/adsorption. Since CTAB is a cationic surfactant, the adsorption of MO could have been facilitated (due to the possible presence of the surfactant on the surface of the semiconductor).

The abbreviation of the samples were supplemented with the word “after” to indicate that they had been used for degradation tests (example: AgBr_HBr_PVP_after). In the XRD patterns, the @ symbol marks the newly formed materials after the degradation tests, while the # symbol marks those compounds that were present before the degradation tests.

The materials' stability was investigated by recycling tests using two different approaches: (i) sequential method, where the MO concentration was readjusted by the addition of MO from the concentrated stock solution; (ii) regenerated catalysts method, where the catalyst was washed with 3× ≈50 mL of H_2_O for 10 min at 4400 rpm and dried for 12 h at 40 °C between the two degradation processes. The protocol for the stability tests mentioned above was the same as the “main” photocatalytic tests, except the sampling intervals were changed to 30 minutes.

X-ray photoelectron spectroscopy (XPS) measurements were recorded with a Specs Phoibos 150 MCD system equipped with a monochromatic Al-Kα source (1486.6 eV) at 14 kV and 20 mA, a hemispherical analyzer, and a charge neutralization device. The catalyst samples were fixed on a double-sided carbon tape where the powder completely covered the tape. The binding energy scale was charge referenced to C 1s at 284.6 eV. High-resolution Ag 3d, Br 3d, S 2p, and C 1s spectra were obtained using an analyzer pass energy of 20 eV in steps of 0.05 eV. Data analysis was carried out with the CasaXPS software.

The relation between the structural, optical, and morphological properties of the obtained samples and their degradation yields after 1 and 2 hours were analyzed using generalized linear models. Two models were constructed using degradation yield percentages as dependent variables and all the measured properties as independent variables. The final models were obtained after a backward stepwise model selection, eliminating the independent variables with the highest probability value in each step until the model contained only independent variables with probability values lower than 0.1. Statistical analysis was carried out using the R 3.1.1 Statistical Environment.

## Results and discussion

### The proposed research plan

As has already been detailed in the introduction, the effect of surfactants/capping agent (PVP, SDS, CTAB) and the S1 chemical elements could be essential as the morpho-structural properties and photocatalytic activities could be affected by the nature of the precursors and the shape-tailoring agents. The reason for using different Br sources was mainly to investigate the effects of different radii. However, these shape-tailoring agents are among the most researched items applied in the synthesis of photocatalytic materials.

**Fig. 1:**
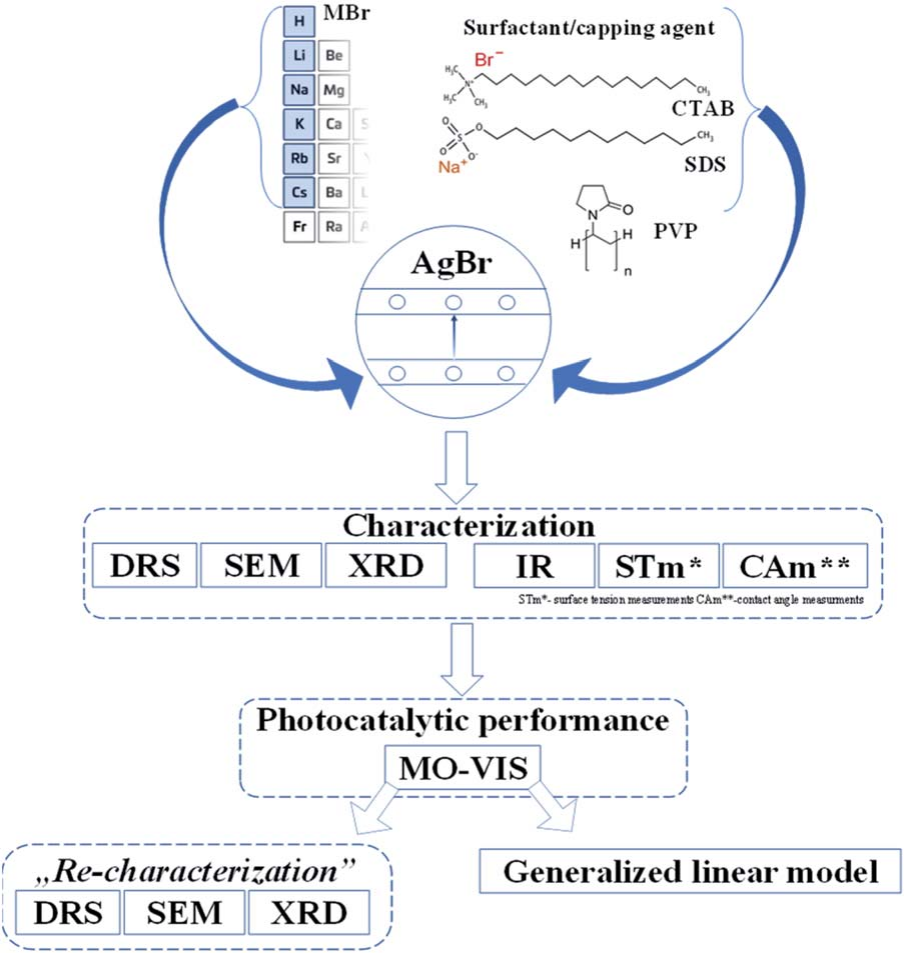
Schematic diagram of the applied research strategy.

Moreover, the comparative investigations using cationic (CTAB), anionic (SDS), and non-ionic (PVP) surfactants/capping agents could give information about how the morphology, the photocatalytic efficiencies, and the reusability could be affected by the nature of these agents (Fig. 1).

After performing the afferent morpho-structural, optical, and photocatalytic measurements, some of the characterization methods (Fig. 1) were repeated on the previously used materials. A correlation between the results was established with the generalized linear model, taking into account the transformations occurring on the surface of the catalysts and the as-obtained photocatalytic efficiencies.

### Structural characterization of the AgBr catalysts

X-ray diffractometry (XRD) was used to determine the crystal structure of the samples and to investigate the effect of the applied surfactants on the (111)/(200) and (220)/(200) crystallographic plane ratios.

**Fig. 2:**
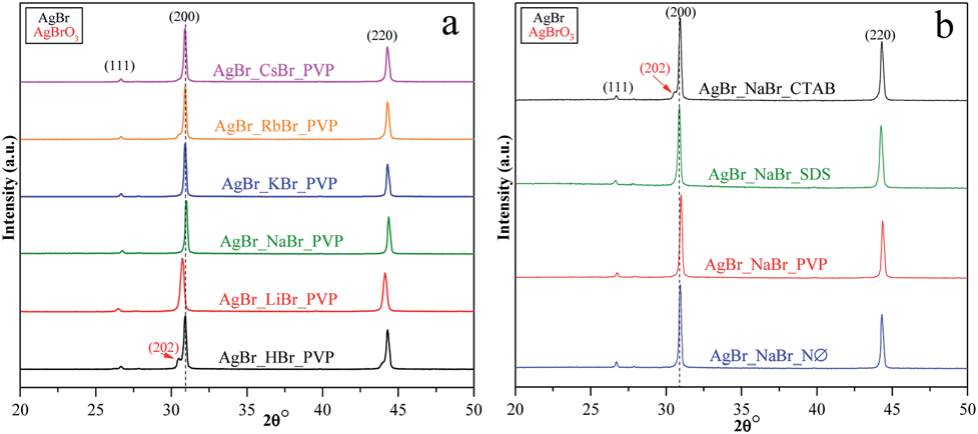
XRD patterns of AgBr photocatalysts prepared (a) with different alkali metals (Li^+^, Na^+^, K^+^, Rb^+^, and Cs^+^) and H^+^ together with PVP; (b) with different surfactants/capping agent using NaBr as the bromide source.

The XRD measurements revealed that face-centered cubic crystals were obtained with diffraction peaks of AgBr located at 26.6 (2*θ*°, (111)), 30.8 (2*θ*°, (200)), and 44.2 (2*θ*°, (220)) (COD card no. 00-150-9151) (Fig. 2, S2a, c, and e†). In the XRD patterns, no specific diffraction peaks of Ag nanoparticles were detected; even so, AgBr is generally considered unstable.^35^ Therefore, we can conclude that the synthetic conditions did not favor the formation/deposition of Ag nanoparticles.

**Fig. 3:**
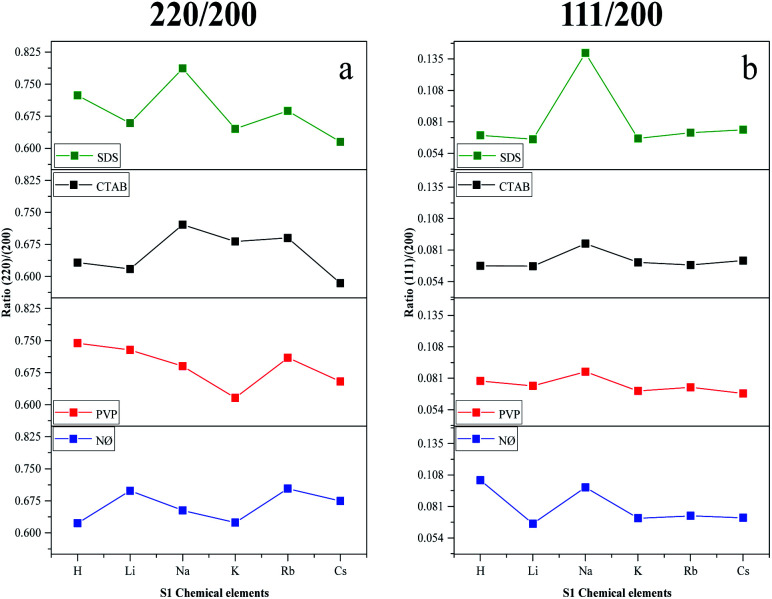
Effect of the alkali metals (Li^+^, Na^+^, K^+^, Rb^+^, and Cs^+^) and H^+^ together with different surfactants/capping agents; diffraction ratio of (a) (220) and (200); (b) (111) and (200).

We have also determined the ratios between the (220)/(200) and (111)/(200) crystallographic planes (Fig. 3). Two similar trends could be observed between the PVP and NØ sample series and between CTAB and SDS by analyzing the intensity of the (220)/(200) ratio, respectively.

In polycrystalline AgBr samples (COD card no. 00-150-9151), the ratio between (220) and (200) is 0.69. In some samples (Table 1, *e.g.*, Cs^+^ and K^+^ series), a lower ratio was obtained, which resulted from the increased amount of the (200) crystallographic plane. This phenomenon is already known^50^ and was attributed to the stabilizing effect of Br^−^ on the (200) crystallographic plane. Therefore, it can be presumed that the concentration of Br^−^ influenced the ratio between the (111) and (200) planes. The intensity of (111)/(200) varied similarly in the NØ-, CTAB-, and PVP-based samples.

**Table 1:** Structural, optical, and morphological properties, surface tension, contact angle values, and the degradation yields of AgBr-based materials

Sample	*d*_XRD_ (nm)	Ratio (111)/(200)	Ratio (220)/(200)	*d*_SEM_ (μm)	Band-gap energy (eV)	Contact angle value (°)	*σ* (mN m^−1^)	Degradation yield after^b^ (%)	
								1 hour	2 hours
AgBr_HBr_NØ	n.a.^a^	0.103	0.622	2.89	2.37	59.3	53.92	52.0	85.0
AgBr_HBr_PVP	n.a.^a^	0.078	0.743	1.03	2.37	51.2	53.87	84.1	88.1
AgBr_HBr_CTAB	36.8	0.067	0.632	3.85	2.40	n.a.^a^	51.56	23.5	43.9
AgBr_HBr_SDS	36.8	0.069	0.723	3.15	2.40	n.a.^a^	52.81	41.0	51.1
AgBr_LiBr_NØ	39.5	0.066	0.698	3.02	2.41	n.a.^a^	46.86	64.4	71.25
AgBr_LiBr_PVP	30.5	0.074	0.728	0.47	2.33	32.6	50.28	**90.6**	**91.9**
AgBr_LiBr_CTAB	34.0	0.067	0.617	3.79	2.38	n.a.^a^	46.40	65.5	83.8
AgBr_LiBr_SDS	39.5	0.065	0.658	3.15	2.34	n.a.^a^	49.53	41.8	69.7
AgBr_NaBr_NØ	42.1	0.097	0.652	3.04	2.32	64.5	52.90	52.1	80.8
AgBr_NaBr_PVP	40.7	0.086	0.690	0.75	2.29	46.9	50.67	83.8	84.3
AgBr_NaBr_CTAB	36.6	0.086	0.721	4.29	2.43	86.3	48.72	87.6	91.2
AgBr_NaBr_SDS	39.7	0.140	0.786	3.67	2.37	n.a.^a^	51.80	38.3	60.1
AgBr_KBr_NØ	34.2	0.070	0.623	3.06	2.40	n.a.^a^	47.66	47.9	77.5
AgBr_KBr_PVP	33.8	0.070	0.615	0.98	2.40	53.4	50.63	84.2	84.8
AgBr_KBr_CTAB	42.3	0.070	0.681	3.19	2.40	46.8	45.93	38.8	53.0
AgBr_KBr_SDS	33.3	0.066	0.645	3.03	2.38	n.a.^a^	45.94	59.5	83.3
AgBr_RbBr_NØ	36.4	0.072	0.703	3.25	2.38	n.a.^a^	52.95	37.8	54.9
AgBr_RbBr_PVP	n.a.^a^	0.073	0.709	1.84	2.38	52.8	51.88	67.1	85.0
AgBr_RbBr_CTAB	38.1	0.068	0.690	3.55	2.44	n.a.^a^	51.81	10.4	43.9
AgBr_RbBr_SDS	40.0	0.071	0.687	2.94	2.34	n.a.^a^	48.84	21.0	52.7
AgBr_CsBr_NØ	40.6	0.071	0.674	3.26	2.48	63.3	48.01	57.9	86.6
AgBr_CsBr_PVP	37.9	0.067	0.654	0.38	2.32	37.7	48.09	80.4	88.7
AgBr_CsBr_CTAB	39.1	0.071	0.584	3.27	2.38	88.6	48.98	0.0	0.0
AgBr_CsBr_SDS	35.4	0.074	0.615	3.31	2.43	61.4	49.06	54.3	81.3

a n.a. – not available.

b Bold values mark the highest degradation values.

It seems that the appearance of the (111) crystallographic plane is independent of the metal ions present in the synthetic mixture. The AgBr_NaBr_SDS sample had the highest ratio of (111)/(200) (Fig. 3b), which was also visible in the SEM micrographs, where polyhedral structures were observed (section of Morphological investigations (SEM); Fig. 6).

The highest ratio values were achieved using PVP, resulting in a more pronounced presence of the (111) crystallographic plane, which is essential in photocatalytic processes.^34^

In the case of AgBr_RbBr_PVP and AgBr_HBr_PVP (Fig. 2a), a small amount of AgBrO_3_ was also detected (COD card no. 00-101-0507), which is also considered to be a photocatalyst.^51,52^ The specific diffraction peaks of AgBrO_3_ overlapped with the 30.8 (2*θ*°, (200)) diffraction peak of AgBr. Despite the assumption that the AgBr/AgBrO_3_ system can act as an efficient photocatalyst, it has already been demonstrated in the literature that under visible light irradiation, it inevitably transforms into Ag/AgBr.^52^ The formation of AgBrO_3_ was also observed in AgBr_HBr_NØ (Fig. S2a†) and AgBr_NaBr_CTAB (Fig. S2e†).

### Optical properties (DRS) and surface-anchored organic groups (IR spectroscopy)

**Fig. 4:**
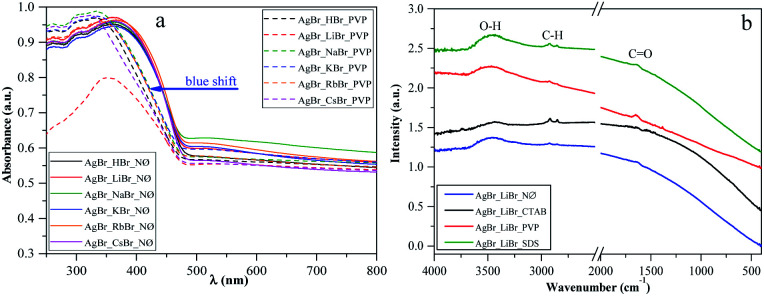
(a) DRS of silver halides obtained in the presence of different alkali metals (Li^+^, Na^+^, K^+^, Rb^+^, and Cs^+^) and H^+^ together with PVP as a capping agent *vs.* the reference NØ samples, and (b) IR spectra of AgBr_LiBr with different surfactants/capping agent (NØ; CTAB; PVP; and SDS).

One of the main determining factors of the photocatalytic activity is the structure of the electronic bands, which can be characterized by the band gap energy ((Table 1), calculated using the Kubelka–Munk approach^48,49^). We did not find any specific plasmonic resonance bands of Ag nanoparticles (Fig. 4a; S3a and b†). This is the second proof that the as-prepared silver bromides are stable (the first one is the corresponding XRD patterns, Fig. 2; S2a, c and e†).

Considering the results obtained using the S1 chemical elements, we observed that using K^+^, the obtained band gap energy values were ≈2.40 eV for each sample.

Moreover, using different surfactants/capping agents, we focused on two groups of cations. They were divided according to their ionic radius as follows: H^+^, Li^+^, and Na^+^ were considered as cations with “small” ionic radius, while K^+^, Rb^+^, and Cs^+^ were considered as cations with “large” ionic radius. The obtained dependencies were as follows (Table 1):

- CTAB and NØ samples showed opposite trends. In the case of the NØ series, the trend of the dependence of the used cation on the applied bromide sources was Li > H > Na and Cs > K > Rb (similar to the SDS series), while for the CTAB series, it was Na > H > Li (as in the case of SDS) and Rb > K > Cs.

- Using PVP, the unique trends H > Li > Na and K > Rb > Cs were obtained, with generally lower band gap energy values. The lowest value was obtained for AgBr_NaBr_PVP (2.29 eV; Table 1), which could also be in correlation with the highest intensity ratio of (111)/(200) (0.074; Table 1). Therefore, the usage of PVP influenced the band gap energy of the catalysts.

According to the XRD patterns, we found the same trend for the CTAB samples when the (220)/(200) intensity ratio and the band gap energy values were considered.

It should be noted that AgBrO_3_ was not identified in the DRS spectra of the samples, including the first-order derivative of the spectra (no specific electron transition bands were observed).

However, in the PVP series, a blue shift of the light absorption edge was noted (Fig. 4a), which could be originated from the residual surface-anchored PVP.^53^ To reinforce this finding, IR spectroscopy measurements were carried out. Moreover, the smaller particle size of this group of samples could also be an explanation for this behavior. To clarify this, the morphological aspects will be further discussed in the section dealing with Morphological investigations (SEM).

The specific absorption peaks observed in the IR spectra (Fig. 4b) were assigned to –C=O (1641 cm^−1^), –CH_3_, –CH_2_ (2974 cm^−1^, 2848 cm^−1^), and O–H (3500 cm^−1^) stretching vibrations. The red-shifting of the specific –C=O band can also be observed, which can be correlated with the fact that PVP is coordinated through –C=O groups with the silver atoms. In the NØ series, sample-specific bands for O–H and –CH_3_, –CH_2_ were also present, which could serve as the proof that EG was anchored on the surface.

### Contact angle measurements

**Fig. 5:**
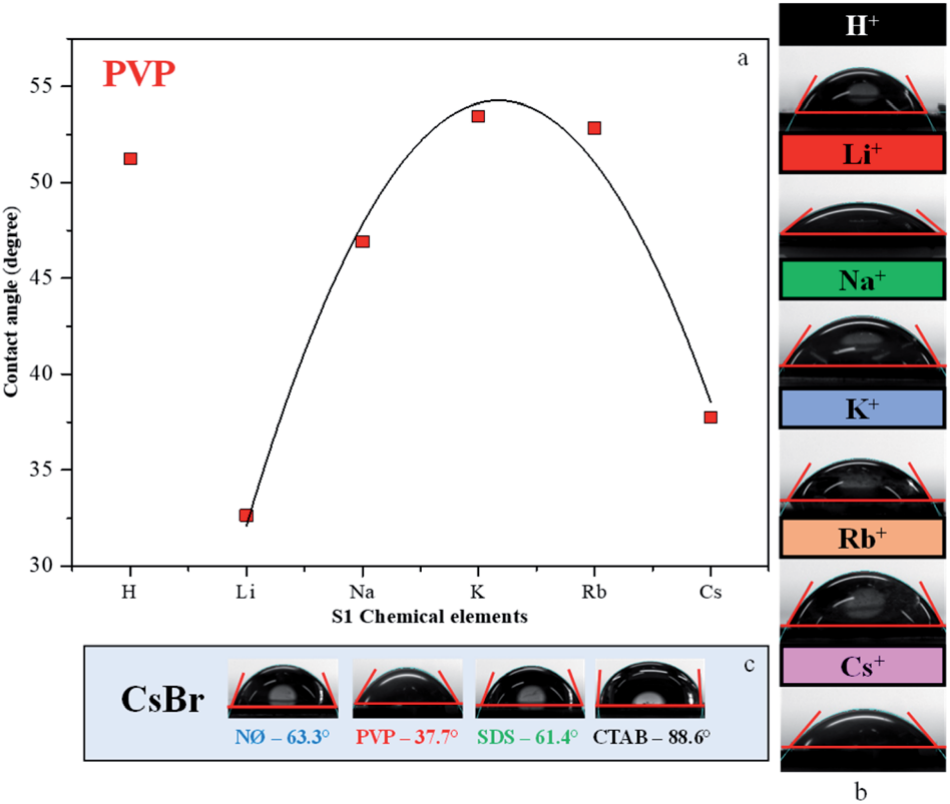
Contact angle measurement of AgBr: (a) the dependence between the contact angle values and different alkali metals (Li^+^, Na^+^, K^+^, Rb^+^, and Cs^+^) and H^+^ together with PVP; the contact angles of (b) PVP-modified samples and (c) CsBr sample series with different surfactants/capping agent.

Generally, high hydrophilicity is a requirement for an efficient photocatalytic process; thus, the interaction between the catalyst and water was examined. The influence of S1 chemical elements on the contact angle values was investigated (for the samples obtained in the presence of PVP). We observed that the AgBr_LiBr_PVP (32.6°) and AgBr_CsBr_PVP (37.7°) samples showed the lowest contact angle values, while the others were between 46.9–53.4° (Table 1 and Fig. 5a–c). The PVP-modified samples were more hydrophilic in comparison with the other samples. Therefore, the suspendability of the materials (in aqueous media) can be attributed to the adsorbed PVP (Fig. 4b). This behavior can be explained by the fact that in the NØ sample series, the system did not contain any added surfactants, while, in the case of SDS samples, the surfactant could be easily removed during the cleaning process.

The samples containing CTAB (Fig. 5c) generally showed the highest contact angle value, which was unusual. In previous investigations, it was claimed that it could be due to the formation of micelles^54^ or due to the non-development of micelles.

### Surface tension of the solutions containing the shape-tailoring agents and alkali metal salts

The compounds that were used during the synthesis influenced the hydrophilicity of the catalysts as was confirmed before. Thus, we have investigated the effect of the surfactants on the surface tension values of the synthetic solution A (section of Solvothermal synthesis of AgBr photocatalysts) to explain the origins of the obtained properties. The surface tension value obtained for pure EG is 49.79 mN m^−1^, which was, in this case, the absolute reference. Considering the S1 chemical elements, we have observed that the Cs^+^-modified sample series resulted in approximately the same surface tension values (48.01–49.06 mN m^−1^, Table 1) independently of the used surfactant. When HBr was used as the bromide source, the surface tension values were higher than that of pure EG, which were independent of the used surfactant.

Furthermore, we have found that no specific trends could be observed using different surfactants, both for SDS and CTAB. Meanwhile, for PVP, the surface tension measurements resulted in the same values (Table 1). We can generally conclude that the surface tension value was not affected by the character of the applied surfactants/polymer.

Using CTAB, the growth of the (220) plane was favored. This fact links the surface tension directly with the obtained microcrystals' geometry. Therefore, we can conclude that for the growth of the (111) plane, SDS and PVP mainly were responsible.

### Morphological investigations (SEM)

In order to examine the morphology of the AgBr-based samples, the SEM micrographs were recorded. It was observed that using PVP, polyhedral structures were formed on the microcrystals, which can enhance the photocatalytic activity.^55^ We did not find any clear correlation between the used S1 chemical elements and the obtained average particle size (Table 1).

**Fig. 6:**
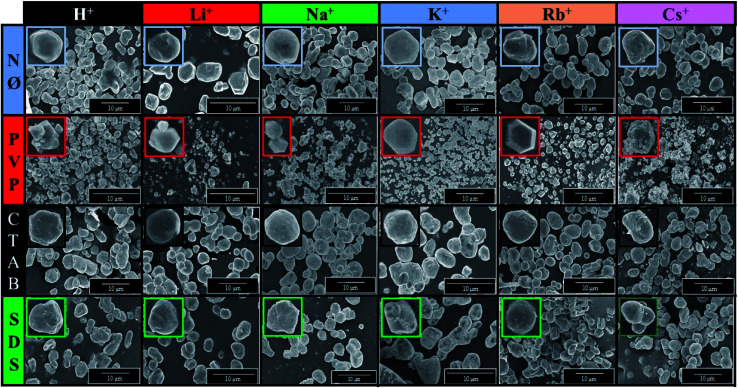
SEM micrograph series of AgBr photocatalysts prepared using different alkali metals (Li^+^, Na^+^, K^+^, Rb^+^, and Cs^+^) and H^+^ and surfactants/capping agent (NØ, PVP, CTAB, and SDS).

Furthermore, considering the applied surfactants/capping agent, the following observations were made after analyzing the morphology of the samples:

- Using PVP, the degree of monodispersity (Fig. S4†) was higher, which was within the range of 0.38–1.84 μm. The highest monodispersity was registered for AgBr_LiBr_PVP (Fig. 6, S4 and Table 1) with an average particle size of ≈410 nm. With the increase in the ionic radii of the cations, the monodispersity of the samples decreased, culminating in the case of Cs^+^ (0.5–3 μm sized particles were formed, as shown in Fig. S4†).

- In the case of AgBr_RbBr_PVP and AgBr_CsBr_PVP samples, larger aggregates were observed (Rb^+^: ≈2 μm; Cs^+^: ≈4 μm) with some smaller crystals (0.4–0.7 μm) as well. Wang *et al.*^27,28^ also concluded that microcrystals with a polyhedral structure could be obtained using PVP as the surfactant. PVP influenced the formation of the (111) crystallographic plane, which was responsible for the polyhedral morphology. This influence was also proved in the section dealing with the surface tension of the solutions.

- In the case of NØ, the particles did not have any specific shape (Fig. 6). It is not surprising that the different cation ion radii did not have any apparent effect on the catalysts' morphology as a non-specific trend was also observed in the case of the surface tension values of the synthetic solution A (containing the shape-tailoring agents and the alkali metal salts).

- Using anionic (SDS) and cationic surfactants (CTAB), quasi-spherical (Fig. 6) microcrystals were obtained.

It is worth mentioning that an apparent discrepancy was observed between the particle sizes obtained by XRD (using the Scherrer equation) and SEM. This suggests that a hierarchical build-up occurred during the synthesis as the primary crystallites with dimensions in the range of 30–42 nm were aggregated to particles with dimensions between 0.35–4.63 μm (Table 1).

### Degradation of methyl orange under visible light

The reasons for using MO as the model pollutant and visible light source are presented in ESI (Fig. S5†).

**Fig. 7:**
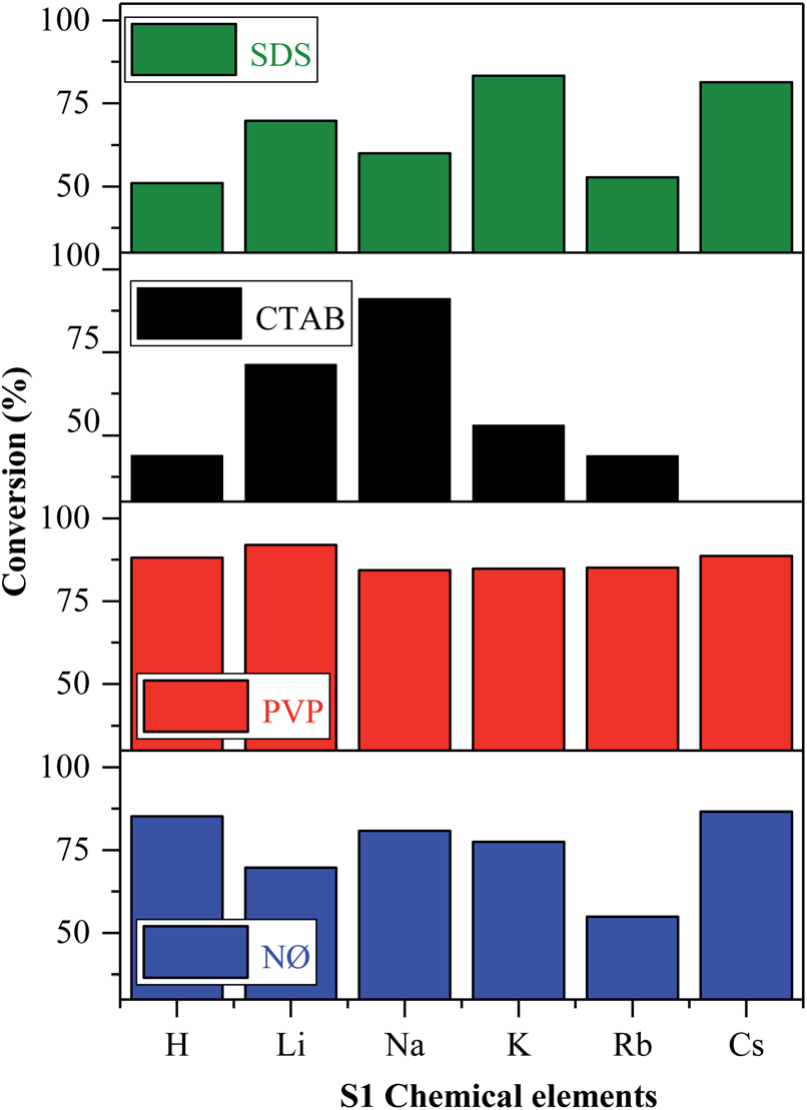
Photocatalytic degradation of MO in the presence of AgBr under visible light irradiation.

According to the mechanism suggested by Kuai *et al.*,^22^ the Ag nanoparticles formed *in situ* on the surface of AgBr, while Br^−^ was oxidized to Br^0^, which could interact with the model pollutant. The oxidation of Br^−^ to Br^0^ was visible in our case, while we did not find any evidence of elemental bromine formation. This finding will be further discussed in the section dealing with the Stability investigation of the AgBr_LiBr_PVP sample based on the results obtained by XPS. As shown in Fig. 7, all the synthesized catalysts showed noticeable photocatalytic activity towards methyl orange, except for AgBr_CsBr_CTAB, which showed high adsorption capacity.

Thus, the question arises whether the achieved removal was adsorption or degradation. Therefore, IR measurements (Fig. S6†) were carried out to clarify this issue.

During the measurements, the detected bands were as follows. The band at 1384 cm^−1^ can be attributed to N=N vibrations. The band at 1250–1000 cm^−1^ is due to the presence of sulfonate species, which did not accumulate during the degradation process. Based on these results, it can be concluded that in our case, degradation indeed took place (Fig. S6†).

In the case of the other S1 chemical elements, *i.e.*, H^+^, K^+^, Rb^+^, and Cs^+^-, the same trend was observed and the following observations were made (Fig. 7):

- The sample series based on HBr resulted in the same activity trend as the surface tension values.

- In the case of the LiBr and RbBr sample series, the obtained conversion trend is similar to the intensity ratio change of the (111)/(200) crystallographic planes (Fig. 3b).

Furthermore, using different surfactants/capping agent, the following observations were made:

- The highest conversion values were obtained using the materials synthesized in the presence of PVP. The following conclusions/explanations can be deduced from the obtained results:

(i) The PVP samples showed the lowest contact angle values (Fig. 5a and b), indicating the higher hydrophilicity;

(ii) They had the lowest band gap energy values (Table 1) compared with the used different alkali salt cation radii and surfactants (exceptions: AgBr_RbBr_SDS and AgBr_KBr_SDS);

(iii) The ratio of the (111)/(200) plane was the highest (Fig. 3b) in the case of the PVP-modified samples, which correlate with the morphology of the samples.

- The adsorption of MO occurred in the case of the CTAB-modified sample series. The lowest degradation yield was obtained when CTAB was used. In the case of AgBr_HBr_CTAB, AgBr_KBr_CTAB, and AgBr_RbBr_CTAB, the conversion values barely reached 38.7% (Table 1) after one hour. After the second hour, only half of the MO was degraded. The lower degradation values could be attributed to the highest contact angle values (Fig. 5c). The AgBr_NaBr_CTAB sample showed the highest degradation yield compared with the other CTAB samples from the series, which can be attributed to the highest ratio of the (111)/(200) crystallographic planes' intensity (Fig. 3b). In the case of CTAB, we can also conclude that a volcanic-type trend was obtained and the maximum was observed in the case of sodium (Fig. 7).

- In the case of the NØ and SDS sample series, we did not find any obvious correlation compared with the other parameters.

Fig. S7† presents the degradation curves of the most efficient samples. However, it was interesting to note that the lower band gap energy values did not positively influence the degradation yields. To reinforce the correlations, mathematical approaches were used to validate the results.

Using generalized linear models (summarized in Table 2), we found that the primary crystallite size values (calculated by the Scherrer equation, Table 1) had a significant negative effect on both the degradation yields (after 1 and 2 hours). Moreover, the same effect could be observed in the surface tension values (calculated by the equation described in section Characterization of the methods and instrumentation) only after 1 hour. However, the intensity ratio of the (111) and (200) crystallographic planes (Table 1) had a significant positive effect on the degradation yield after 1 and 2 hours (Table 1) as well. The negative effect of the primary crystallite size could be attributed to the fact that smaller particles usually result in higher photocatalytic activities.^56^ Moreover, the primary crystallite size values can be directly linked to the surface tension values, *i.e.*, lower surface tension values could easily yield smaller crystals as was observed numerous times during the application of different surfactants for the synthesis of nanoparticles.^57^ On the other hand, the intensity ratio of the (111) and (200) crystallographic planes could have a positive effect due to their polyhedral structure (Fig. 6), which results in a higher photocatalytic activity.^55^

**Table 2:** The effect of the structural properties of the samples on their degradation yields after 1 and 2 hours (*t* = *t* value; *p* = probability; *p ≤ 0.05; **p ≤ 0.01; ***p ≤ 0.001)

		*t*	*p*
Degradation yield (%) 1 hour	*d*_XRD_ (nm)	−2.764	0.015*
	Ratio (111)/(200)	2.390	0.032*
	*σ*_solution A_ (mN m^−1^)	−2.310	0.036*
Degradation yield (%) 2 hours	*d*_XRD_ (nm)	−2.247	0.041*
	Ratio (111)/(200)	2.186	0.046*
	*σ*_solution A_ (mN m^−1^)	−2.128	0.051

It should be mentioned that the AgBr_CsBr_CTAB sample was excluded from the statistical analysis due to its extremely high adsorption capacity before the degradation process. Moreover, the AgBr_NaBr_SDS sample was also excluded because it showed very peculiar characteristics.

Different parameters and photocatalytic activities are interdependent on each other, as shown before. Therefore, the next step was to investigate the changes in the catalysts' structure after degradation.

### Analyzing the samples after the degradation processes

At the end of the photodegradation process, we noticed that the pH value of the MO solution changed mostly from 7 to 5 and the color of the catalysts changed from green/greenish-yellow to purple. Considering that this could be attributed to the deposition of silver (Ag^0^)/or silver(i) oxide during the photodegradation process, we further investigated the materials' morpho-structural and optical parameters after the degradation processes using XRD, DRS, and SEM.

**Fig. 8:**
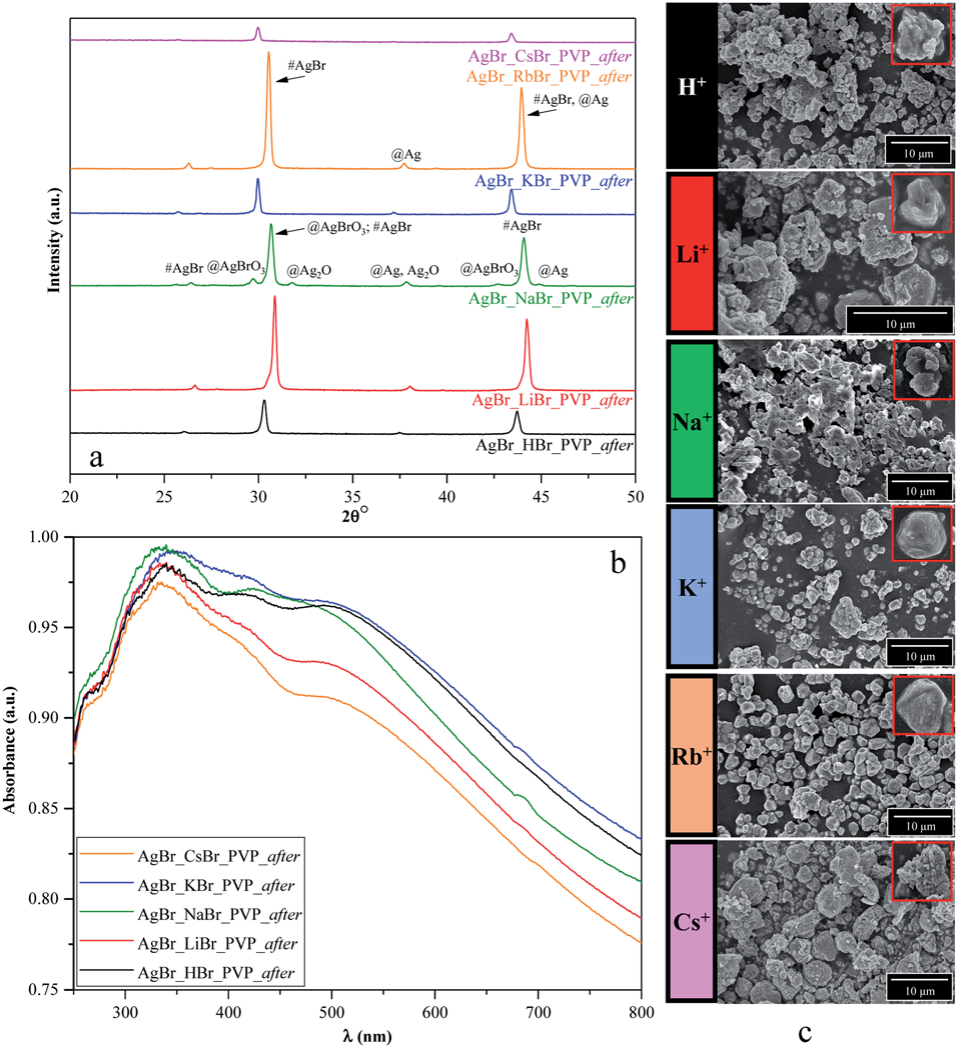
Results of the (a) structural (XRD), (b) optical (DRS), and (c) morphological investigations (SEM) of PVP-modified samples following the degradation process.

As shown in Fig. 8, S2b, d, f, and S3c,† the structure, morphology, and optical parameters of the materials changed following the photocatalytic processes. We presumed that the degradation pathway was correlated with the morpho-structural changes on the samples' surface.

From the point of the surfactants/capping agent, the following observations were made:

(i) PVP based samples

- Based on Fig. 2a, we noticed that the two different samples were AgBrO_3_/AgBr composites (namely, AgBr_HBr_PVP and AgBr_RbBr_PVP); however, after/during the photocatalytic degradation, the specific reflection of AgBrO_3_ disappeared. Simultaneously, Ag signals were detected in the XRD patterns (Fig. 8a).

- The formation of Ag nanoparticles was identified based on the XRD patterns (Fig. 8a) in the case of AgBr_KBr_PVP_after and AgBr_LiBr_PVP_after. A small amount of Ag was also observed in AgBr_CsBr_PVP_after, which was also identified in the DRS spectra through the plasmonic resonance band of silver (Fig. 8b). It seems that the excessive deposition of silver nanoparticles can deactivate the catalyst, while in the first hour of the degradation experiment, silver acts as a charge separator, increasing the efficiency of the photoactive agent.

- In the XRD pattern of the AgBr_NaBr_PVP_after sample (Fig. 8a), specific reflections of AgBrO_3_ and Ag_2_O were observed (although they were less prominent). The specific plasmonic resonance band related to Ag_2_O^58^ can be observed in Fig. 8b, next to the specific band of Ag nanoparticles (in the range of 400–500 nm (ref. 59)) and the electronic transitions of metallic Ag^0^ (in the range of 250–330 nm (ref. 60)).

- In the case of AgBr_LiBr_PVP_after and AgBr_NaBr_PVP_after, according to the SEM micrographs (Fig. 8c), we can presume that the crystal structure changed during photocatalytic degradation.

(ii) CTAB-based samples (Fig. S2f†)

- In the case of AgBr_HBr_CTAB_after (Fig. S1f†), Ag deposition was also an issue and the second-lowest degradation yield was achieved.

- Surprisingly, the amount of AgBrO_3_ was the highest in the case of AgBr_RbBr_CTAB_after, which has nearly the same degradation yield as that of the AgBr_HBr_CTAB_after sample.

(iii) SDS-based samples (Fig. S2d†)

- For AgBr_NaBr_SDS_after, AgBr_KBr_SDS_after, and AgBr_LiBr_SDS_after, the degradation resulted in the AgBr/AgBrO_3_ composite, which showed high degradation yields. It needs to be emphasized that the SDS-modified samples did not contain AgBrO_3_ after the synthesis as the AgBr/AgBrO_3_ composite was formed only after the degradation.

- Moreover, it is surprising that from all the 24 samples, only the AgBr_CsBr_SDS_after sample resulted in the formation of AgBrO_3_ with high photocatalytic performance (other samples resulted in Ag or Ag_2_O nanoparticles following the degradation processes).

(iv) Samples prepared without surfactants/capping agent (NØ sample series, Fig. S2b†):

- AgBr_HBr_NØ sample also contained AgBrO_3_ (Fig. S2a†) after the synthesis but it disappeared after degradation (Fig. S2b†) and Ag nanoparticles were formed during the photocatalytic process.

- In the case of AgBr_LiBr_NØ, AgBr started to transform into Ag and AgBrO_3_ during/after the photocatalytic process.

Furthermore, from S1 chemical elements, in the case of the LiBr sample series, all the samples resulted in a mixture of AgBr, AgBrO_3_, and Ag nanoparticles in different quantities. Besides, we can conclude that in all the samples that contained AgBrO_3_ initially, the amount of AgBrO_3_ disappeared and transformed into Ag nanoparticles during the degradation processes.

### Stability investigation of the AgBr_LiBr_PVP sample

**Fig. 9:**
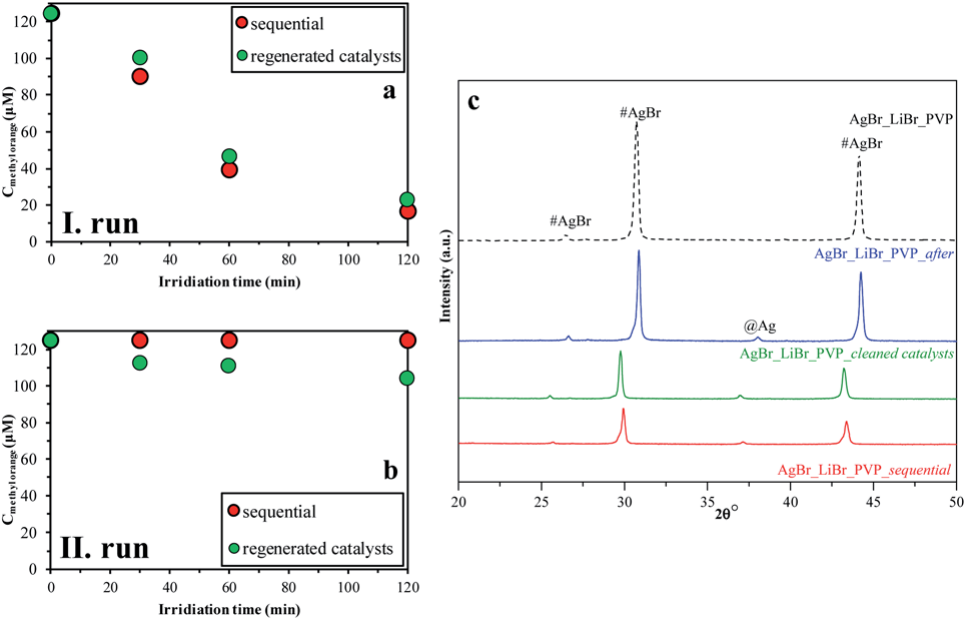
Recycling test on AgBr_LiBr_PVP by two different methods (sequential (red); regenerated catalysts (green)): (a) I run; (b) II run; and (c) their XRD patterns before and after the degradation processes of MO.

In the last step, we analyzed the reusability of the samples by two different methods. For this purpose, the AgBr_LiBr_PVP sample was chosen because it had the highest degradation yield (Table 1). During the degradation processes, the absorption peak related to MO showed a red-shift, which can be due to the protonation of the MO. We can suppose that this is related to the intermediates that were formed during the degradation processes. The results observed in the case of the regenerated catalysts method differ from the ones obtained using the sequential method because the catalysts were cleaned between the two measurements (Fig. 9a and b). By cleaning them, the intermediates could have been washed off from the catalysts' surface, increasing the degradation yields of MO in this way.

After the structural analysis of the catalysts that were measured after degradation (Fig. 9c), we can draw two main conclusions:

- The formation of silver nanoparticles after the degradation was independent of the used recycling method.

- The intensity ratio of the (220)/(200) crystallographic planes changed (Fig. 9c) during the catalytic process. After the first degradation, the ratios of the intensities was 0.78, while at the beginning, it was only 0.72. This change could be attributed to the recrystallization process. Besides, this independence on the used investigation approach of stability, after the second process, the ratio of (220)/(200) crystallographic plane intensities decreased.

**Fig. 10:**
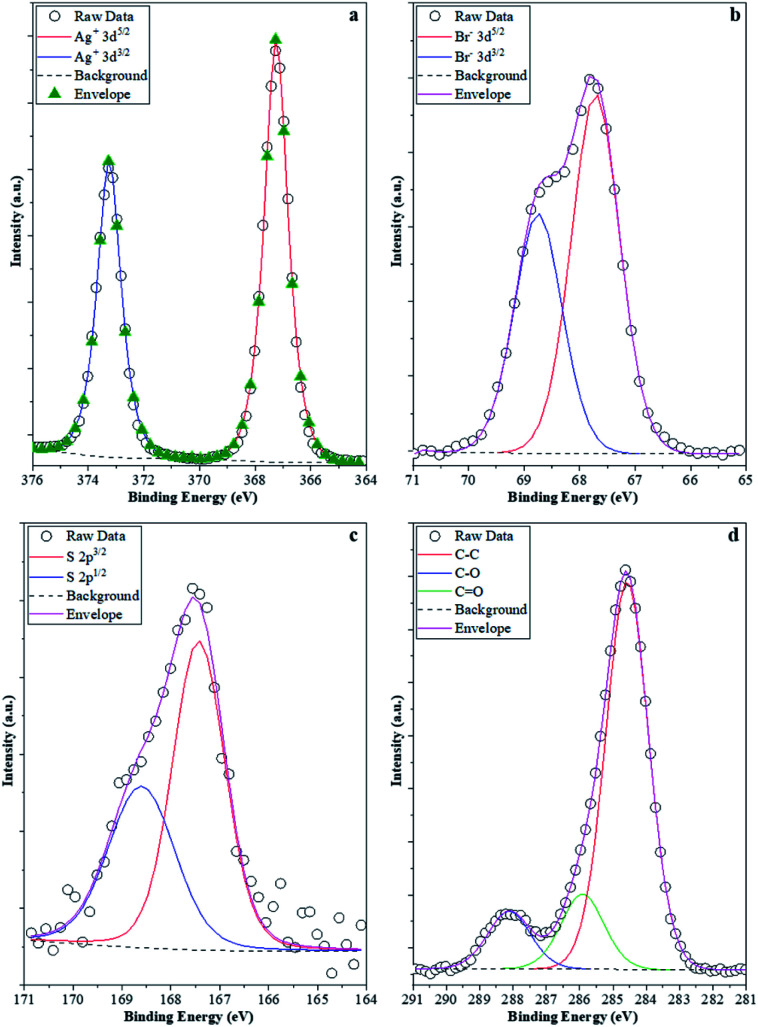
XPS spectra of the samples: (a) Ag 3d; (b) Br 3d; (c) S 2p; and (d) C 1s. Br 3d; (c) S 2p; and (d) C 1s.

The stability investigations showed that significant structural changes occurred during the photocatalytic tests of different AgBr samples. However, these feature changes reflect the properties of the bulk material, while the optical properties could suggest the presence of Ag or Ag_2_O as well. As the investigated processes were taking place on the surface of the photocatalysts, XPS measurements (Fig. 10) were carried out in the case of the four samples (their photocatalytic properties were shown in Fig. 9a and b, their XRD is shown in Fig. 9c, and the partial one of the sample's optical property in Fig. 10).

It was expected that XPS measurements would be capable of demonstrating the possibility of delicate surface-related structural changes of the photocatalyst (AgBr_LiBr_PVP) before and after the degradation processes. Hence, all these elements that were of major interest were investigated. Ag (Fig. 10a) was the first choice as it is known that all silver-based compounds can easily produce metallic Ag. However, in our case, in each of the four samples, just Ag^+^ (373.5 eV-3d_5/2_ and 367.5 eV-3d_3/2_) was observed,^61^ which could either be associated with the silver originated from AgBr or Ag_2_O. Metallic Ag can be excluded because:

- the peaks were symmetric, while in the presence of metallic silver, asymmetrical features should be visible;

- no energy-loss-related signals were observed in the higher binding energy side of each spin–orbit component, which is a characteristic of Ag^0^.

The latter scenario is more probable as Ag_2_O forms immediately once small Ag nanoclusters appear on the surface. It should be mentioned that the Ag 3d XPS spectra of the samples prior to and after the degradation process did not show any difference. This suggests that metallic Ag from several samples (AgBr_LiBr_PVP_after, AgBr_LiBr_PVP_sequantial, and AgBr_LiBr_PVP_cleaned catalyst) was located in the bulk or formed during the XRD measurements^62^ from the deposited oxide layer (which could be amorphous, which is probably the reason why it is not visible in the starting material).

The next investigated element was Br. Br^−^ was the only species detected (66.8 eV-3d_3/2_ and 68.0 eV-3d_5/2_, Fig. 10b) in the samples.^63^ Although no bromate was observed in the AgBr_LiBr_PVP sample, the sample series was verified and it turned out that bromate was absent from the sample. Because MO was used as a model pollutant, we investigated if sulfur could be found on the surface of the samples after degradation. Interestingly, after the degradation process, the S 2p XPS spectra (Fig. 10c) of the samples showed signals that are specific to sulfate (168.8 eV-2p_3/2_, 167.5 eV-2p_1/2_). This was expected as S can be oxidized relatively easily, forming an anchored sulfate group on the surface of the catalyst. No signs of sulfides were noticed; therefore, the formation of Ag_2_S (sulfides can be found at 160.8 eV-2p_3/2_) can be excluded as well. On the surface of the catalysts, the C 1s XPS spectra (Fig. 10d) showed that carbon was abundantly present on the surface. At 284.8 eV, C–C bonds were observed, while at 286.0 eV, C–O–C entities were detected, and finally, at 288.5 eV, O–C=O entities were identified. These signals could easily be originated either from PVP, which is a usual capping agent, or from the oxidation of ethylene glycol during the solvothermal process.^64^ However, interestingly, this signal did not disappear after washing and the degradation processes, pointing out two possible scenarios:

(i) the PVP or EG remains/does not degrade on the surface of the photocatalyst;^65^ or

(ii) the degradation products of the mentioned compounds are adsorbed on the surface containing those functional groups that show the previously mentioned signals.

## Conclusions

The present work investigates the photocatalytic activity and stability issues of AgBr materials, which proved to be more complicated than that discussed before in the literature. Also, the variability of the photoactive materials (AgBr, AgBr/AgBrO_3_) indicates that not only the obtained structural or optical parameters but also the synergistic effects influence their activity.

The photocatalytic activity of silver-bromide was fine-tuned using different precursors during the solvothermal synthesis and the effect of the “S1 chemical elements” (Li^+^, Na^+^, K^+^, Rb^+^, Cs^+^, and the corresponding acid – HBr) and surfactants/capping agent (PVP, CTAB, and SDS) on the optical and morpho-structural properties of the photocatalyst were investigated.

We conclude that a clear relationship exists between the application of PVP and the observed higher photocatalytic activities (in comparison with other surfactants). This could be attributed to (i) the appearance of the (111) crystallographic plane (which was also proved by the generalized linear model), (ii) the lower band gap energy values, and (iii) the lowest contact angles values.

The usage of CTAB resulted in quasi-spherical morphologies with relatively low monodispersity and a photocatalytic activity with a volcanic-type trend, culminating in the case of sodium.

Using H^+^, K^+^, Rb^+^, or Cs^+^ resulted in the same trend of photocatalytic activity, without a significant difference between the applied surfactants/capping agent.

During the synthesis of AgBr, the AgBr/AgBrO_3_ composite was obtained in four cases (AgBr_RbBr_PVP; AgBr_HBr_PVP; AgBr_HBr_NØ; and AgBr_NaBr_CTAB), which was transformed into Ag/AgBr during the degradation of MO.

The obtained AgBr microcrystals were transformed into AgBr-, AgBrO_3_-, Ag-, and Ag_2_O-containing composites after the degradation of MO, which did not have a clear influence on the resulting photocatalytic activities.

Compared with the literature (and partially contradicting it), we conclude that during the degradation processes, elemental bromide did not form; thus, the proposed mechanisms for the degradation of MO (by using AgBr) have to be reconsidered.

## Author contributions

Conceptualization, Zs. R. T. and Zs. P.; methodology, J. K.; investigations T. Gy., M. T., M. K.; formal analysis, Zs. Cz. and L. B.; funding acquisition and resources, L. B. and H. K.; writing, Zs. R. T. and Zs. P.; writing – review and editing, T. Gy. and G. K.; supervision H. K. and G. K.

## Conflicts of interest

There are no conflicts to declare.

## Supplementary Material

RA-011-D0RA09144H-s001
