# Supplementary material for: Shape tailoring of AgBr microstructures: effect of the cations of different bromide sources and applied surfactants
Source: RSC Adv. 2021 Mar 9;11(16):9709–20. doi: 10.1039/d0ra09144h (PMC8695391; doi:10.1039/d0ra09144h)
Supplement: RA-011-D0RA09144H-s001 [file RA-011-D0RA09144H-s001.pdf]

Electronic Supplementary Material (ESI) for RSC Advances.
This journal is © The Royal Society of Chemistry 2021

Supplementary Information

Shape tailoring of AgBr microstructures: effect of the cations of different bromide sources and the applied surfactants

Zsejke-Réka Tóth[a,b], Zsolt Pap[b,c,d], János Kiss[a], Lucian Baia[b,e], Tamás Gyulavári[a], Zsolt Czekes[b,f], Milica Todea[b,g], Klára Magyari[b,c], Gábor Kovács[b,c,*], Klara Hernadi[a,h]

a – Department of Applied and Environmental Chemistry, University of Szeged, Rerrich Béla tér 1, HU-6720, Szeged, Hungary;

b – Nanostructured Materials and Bio-Nano-Interfaces Center, Institute for Interdisciplinary Research on Bio-Nano-Sciences, Babeș-Bolyai University, Treboniu Laurian 42, RO-400271, Cluj-Napoca, Romania;

c – Institute of Environmental Science and Technology, University of Szeged, Tisza Lajos krt. 103, HU-6720, Szeged, Hungary;

d – Institute of Research-Development-Innovation in Applied Natural Sciences, Babes-Bolyai University, Fântânele 30, RO-400294, Cluj-Napoca, Romania;

e – Faculty of Physics, Babeș–Bolyai University, M. Kogălniceanu 1, RO-400084, Cluj–Napoca, Romania;

f – Hungarian Department of Biology and Ecology, Babeş-Bolyai University, Clinicilor 5–7, RO-400006, Cluj-Napoca, Romania;

g – Iuliu Hatieganu University of Medicine and Pharmacy, Faculty of Medicine, Victor Babeş 8, RO-400012, Cluj-Napoca, Romania.

h – Institute of Physical Metallurgy, Metal Forming and Nanotechnology, University of Miskolc, 3515 Miskolc-Egyetemváros, Hungary

* Corresponding author: k.gabor84@chem.u-szeged.hu, gkovacs@chem.ubbcluj.ro (G. K.)

Adsorption measurement:

Experimental:

The adsorption measurement was investigated using a 100 mL beaker. The concentration of MO was 125 μM, while the concentration of the suspension was 1 g · L^{-1}. After 15 minutes of ultrasonication (where the suspension formed), the beaker was covered with aluminum foil (for eliminate any light photon). The adsorption measurement was taken 2 hours with continuous stirring. The sampling was in the first 30 minutes in 5-minutes interval, in the second 30 minutes in 10-minutes interval, and in the last 1 hour in 20-minutes interval.

An *Analytic Jena Specord 250 plus* spectrophotometer was used to analyzed to amount of the adsorbed MO.

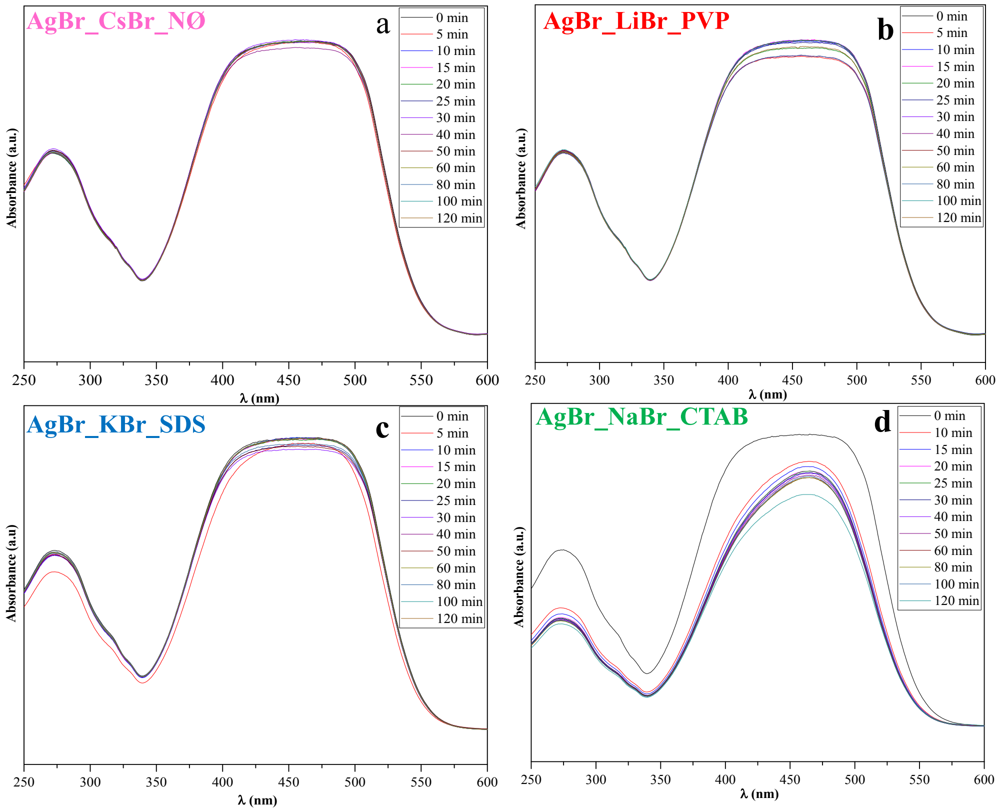

Figure S1: Adsorption curves of AgBr samples: (a) AgBr_CsBr_NØ; (b) AgBr_LiBr_PVP; (c) AgBr_KBr_SDS and (d) AgBr_NaBr_CTAB (model pollutant: methyl orange)

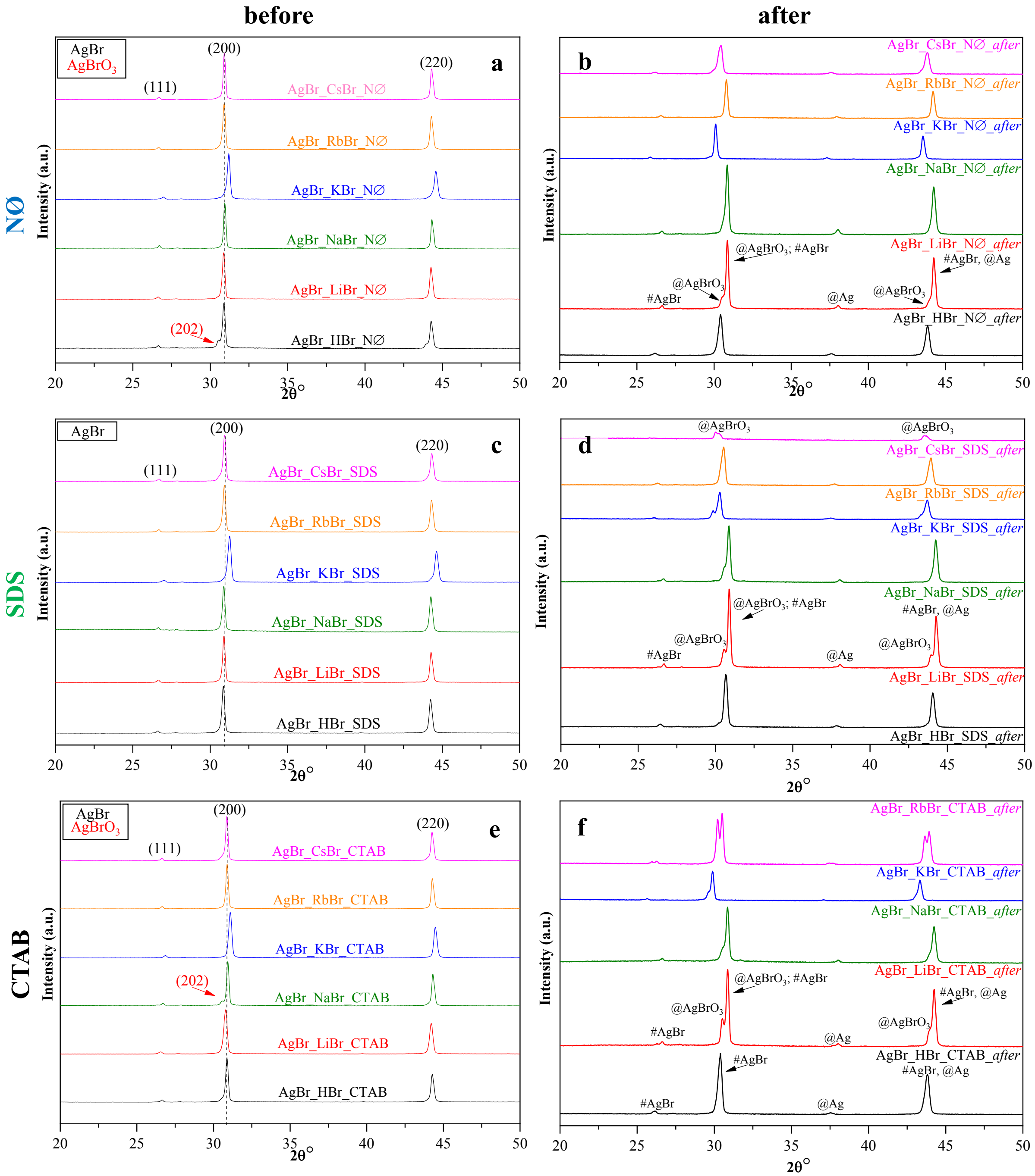

Figure S2: XRD patterns of AgBr photocatalysts prepared with different alkali metals *(Li^+, Na^+, K^+, Rb^+, and Cs^+)* and H^+ obtained (a, b) without surfactant (*NØ*) and by using (c, d) SDS or (e, f) CTAB, (left column) before and (right column) after photocatalytic degradation

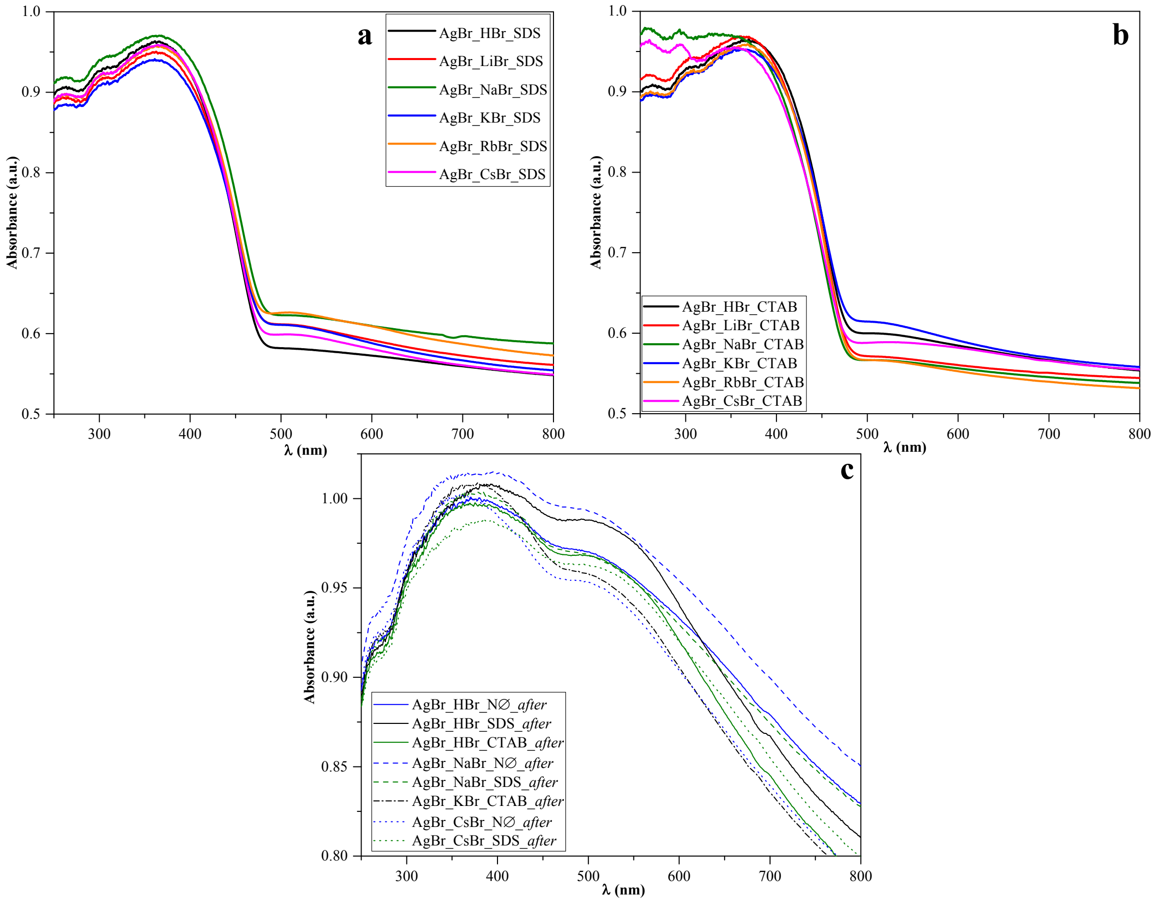

Figure S3: Diffuse reflectance spectra of the silver-halides obtained in the presence of different alkali metals *(Li^+, Na^+, K^+, Rb^+ and Cs^+)* and H^+ obtained with (a) SDS, (b) with CTAB, and (c) *after degradation* process.

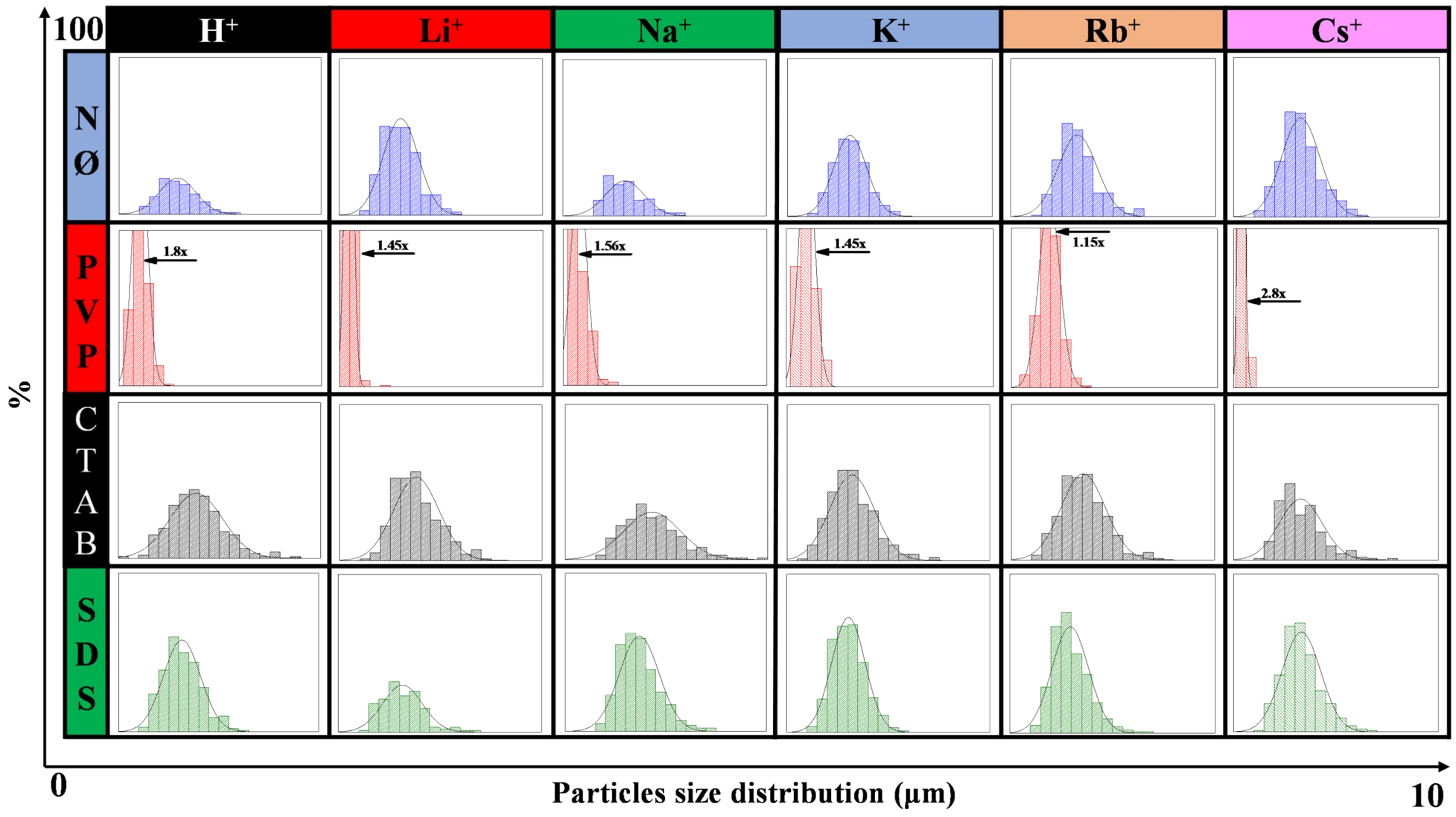

Figure S4: Distribution of AgBr particles sizes based on SEM micrographs from Fig. 6

Selection of the model pollutant:

Material

The phenol degradation was taken under UV light degradation, and the MO degradation was taken under visible (parameters shown in main manuscript) and UV light degradation.

Degradation parameters:

The same equipment was used for UV degradation only the reactor was thermostated by distilled water and irradiated by 6×6 W (λ_{max} = 365 nm) UV lamps. The concentration change of MO was detected same as after visible light irradiation. The decrease in phenol concentration was measured by *Merck-Hitachi L-7100* high-pressure liquid chromatography with *Merck-Hitachi L-4250 UV-Vis* detector ($\lambda_{detection}$ = 210 nm) and a *Lichrospher Rp 18 column* (eluent: 50:50 = MeOH:H_2O).

Results and discussions

In the main article, we have shown that the Br-source and the surfactant can influence the morpho-structural and optical properties of the as-obtained AgBr. Therefore, to investigate the activity differences between these photoactive materials, we have chosen two different model pollutants: i.) phenol, a by-product, and a precursor of the pharmaceutical industry, resulting in more than 28 intermediates through its photocatalytic degradation[1] and ii.) MO, which is a well-known organic dye, used mostly in the textile industry. In the case of MO, we have used two different lamp sources, UV (λ_{max} = 365 nm) and Vis (λ >400 nm), while for phenol degradation, only UV lamps irradiation were applied (generally, the degradation efficiencies of the phenol are lower in visible light irradiation).

For further investigations, we have used AgBr_NaBr based samples. The reason for this selection was that the AgBr_NaBr_PVP and the AgBr_NaBr_NØ had the lowest band gap energy values in comparison with all samples. The AgBr_NaBr_SDS has an abnormal photocatalytic performance, because compared with MO degradation would be excepted a much higher degradation. The reason of the low degradation value can be lies in Ag nanoparticles or other silver-based material deposition. Noticeable, that formation of these materials affected the degradation of model pollutant, but the effect quality (disadvantages or advantages) is questionable. Comparing with the degradation of the methyl orange, the oxidation of the phenol was less efficient (Fig. S4), which can be attributed to the fact that

phenol has a more stable aromatic structure, and it is a poorly adsorbing agent in comparison with MO.

The trend of photocatalytic degradation yields (PVP=CTAB>NØ> SDS) is the opposite, compared to the one observed in the case of (111)/(200) ratio (Fig. 3), causing an increased adsorption rate of the degradation intermediates on the surface, thereby reducing the degradation yield of phenol[2]. Besides, in the literature, methyl orange is more widely used as a model pollutant for testing AgBr (44 vs. 152 articles for phenol and methyl orange, respectively, according to a search on Scopus using AgBr + model pollutant keywords, 26.10.2020).

In the present work, we focused on the photocatalytic degradation using visible light irradiation of methyl orange because the decomposition yields under visible light are higher than the results with UV light irradiation, and this promises them to be used in visible light processes either indoors or outdoors (using the sunlight) while improving their environmentally friendly character.

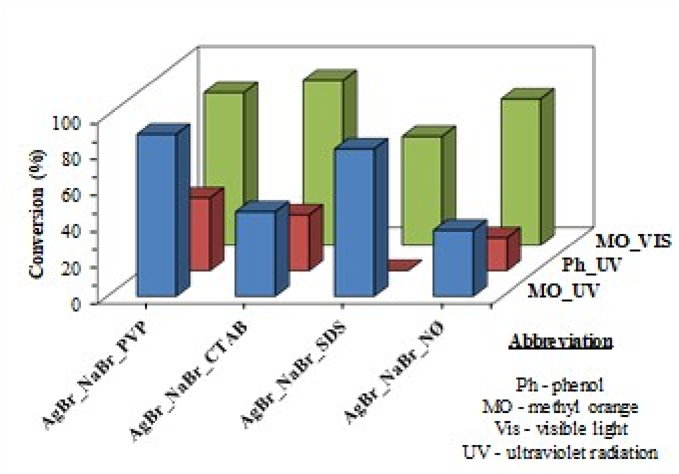

Figure S5: Model pollutant/lamp selection by using AgBr_NaBr photocatalysts

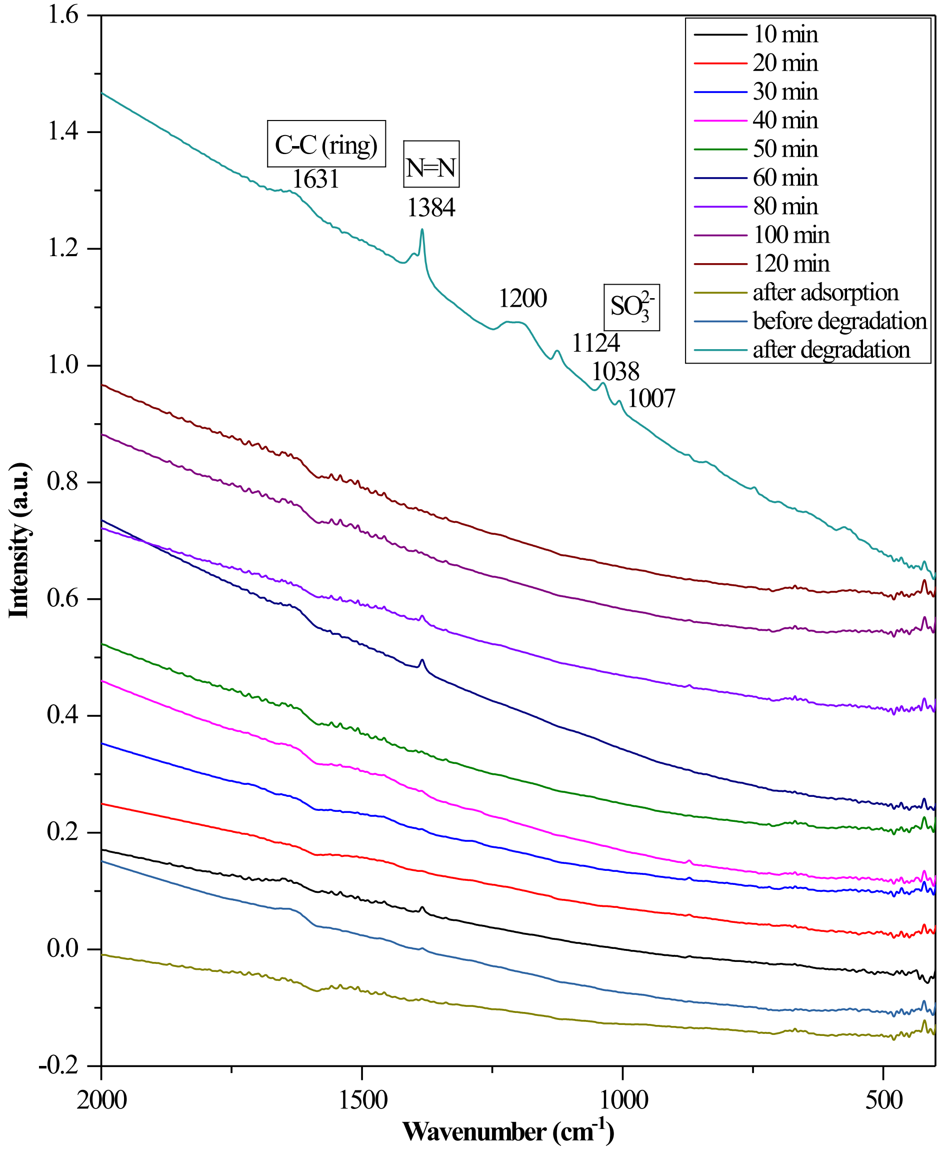

Figure S6: IR spectra of AgBr_NaBr_PVP after degradation processes

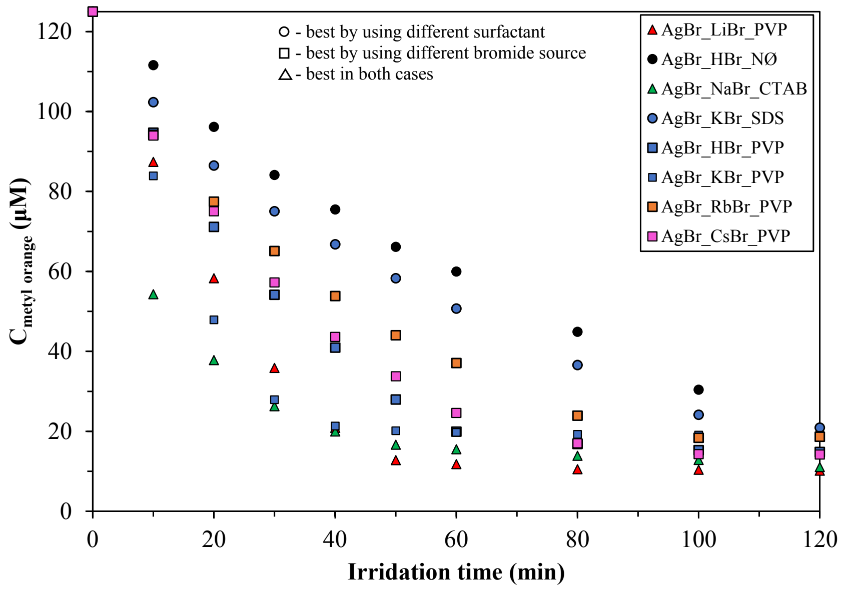

Figure S7: Degradation curve of MO degradation for the most efficient samples.

References:

1. Dang TTT, Le STT, Channei D, Khanitchaidecha W and Nakaruk A. Photodegradation mechanisms of phenol in the photocatalytic process. *Research on Chemical Intermediates*. 2016;**42**(6):5961-74.

2. Tóth Z-R, Kovács G, Hernádi K, Baia L and Pap Z. The investigation of the photocatalytic efficiency of spherical gold nanocages/TiO_2 and silver nanospheres/TiO_2 composites. *Separation and Purification Technology*. 2017;**183**:216-25.
